# Supplementary figures and images for: A Produce Prescription Program in Eastern North Carolina Results in Increased Voucher Redemption Rates and Increased Fruit and Vegetable Intake among Participants
Source: Nutrients. 2022 Jun 11;14(12):2431. doi: 10.3390/nu14122431 (PMC9229115; doi:10.3390/nu14122431)

Produce Prescription Program Flowchart

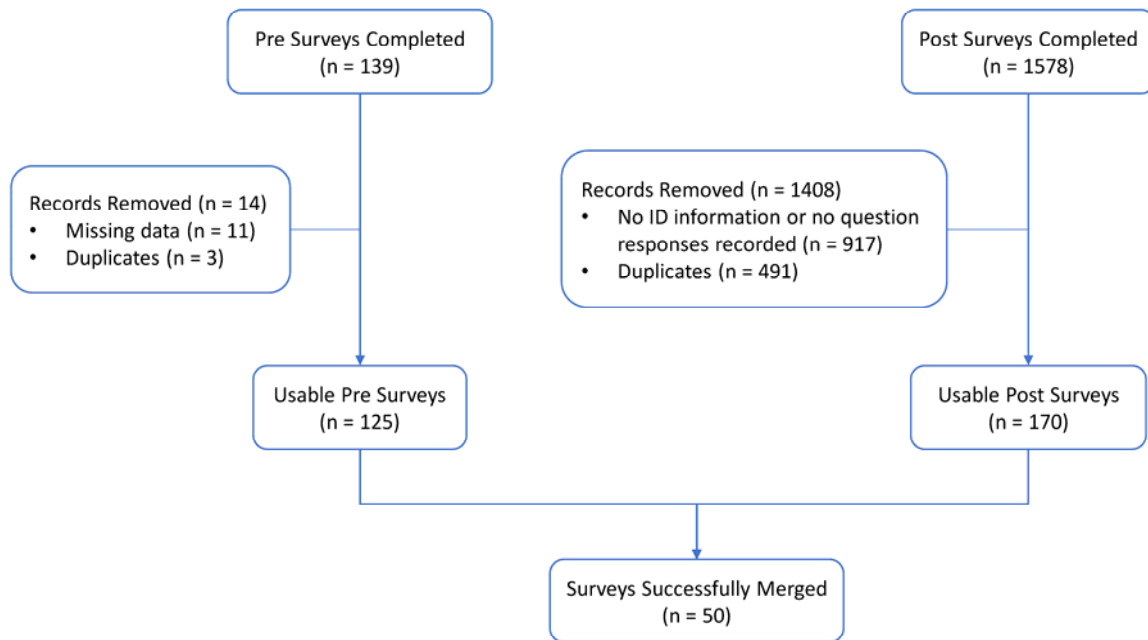

**Figure S1.** Produce Prescription Program Flowchart.

Supplement: Supplementary file 1 [file nutrients-14-02431-s001.zip › nutrients-1728203-supplementary.pdf]
